# Effect of E-64 Supplementation during In Vitro Maturation on the Developmental Competence of Bovine OPU-Derived Oocytes

**DOI:** 10.3390/genes13020324

**Published:** 2022-02-10

**Authors:** Ahmed Z. Balboula, Mansour Aboelenain, Miki Sakatani, Ken-Ichi Yamanaka, Hanako Bai, Takahiro Shirozu, Manabu Kawahara, Abd Elraouf O. Hegab, Samy M. Zaabel, Masashi Takahashi

**Affiliations:** 1 Animal Sciences Research Center, University of Missouri, Columbia, MO 65211, USA; abalboula@missouri.edu; 2 National Agricultural Research Center for Kyushu Okinawa Region, National Agriculture and Food Research Organization, Koshi, Kumamoto 861-1192, Japan; msaka@affrc.go.jp (M.S.); kyama@cc.saga-u.ac.jp (K.-I.Y.); 3 Theriogenology Department, Faculty of Veterinary Medicine, Mansoura University, Mansoura 35516, Egypt; mma247@dls.rutgers.edu (M.A.); abraoshe@yahoo.com (A.E.O.H.); samy.zaabel@yahoo.com (S.M.Z.); 4 Genetics Department, Rutgers University, 145 Bevier Rd., Piscataway, NJ 08854, USA; 5 Institute of Livestock and Grassland Science, National Agriculture and Food Research Organization, Tochigi 329-2793, Japan; 6 Faculty of Agriculture, Saga University, Honjo-machi, Saga 840-8502, Japan; 7 Laboratory of Animal Genetics and Reproduction, Department of Animal Science, Research Faculty of Agriculture, Hokkaido University, Sapporo 060-8589, Japan; hbai@anim.agr.hokudai.ac.jp (H.B.); takahiro.shirozu@fujita-hu.ac.jp (T.S.); k-hara@anim.agr.hokudai.ac.jp (M.K.); 8 Department of Biology, Faculty of Science, Taif University, Taif 26571, Saudi Arabia; 9 Research Faculty of Agriculture/Global Center for Food, Land and Water Resources, Hokkaido University, Sapporo 060-8589, Japan

**Keywords:** ovum pick-up, cathepsin B, E-64, in vitro maturation, developmental competence, bovine oocytes

## Abstract

Recovery of bovine oocytes using the ovum pick-up (OPU) technique offers the advantage of rapid genetic improvement through propagation of desired genes from animals with high genetic qualities. However, the developmental competence of OPU-derived immature oocytes remains relatively poor. We previously found that cathepsin B gene expression and activity are increased in poor quality oocytes and embryos compared to good quality ones. In this study, we investigated the effect of E-64 (cathepsin B inhibitor) supplementation during in vitro maturation (IVM) on the developmental competence of OPU-derived immature oocytes and the quality of the produced blastocysts. Our results showed that supplementation of IVM medium with E-64 significantly improved the developmental competence of OPU-derived immature oocytes as evidenced by the significant increase of the blastocyst rate. Importantly, the presence of E-64 during IVM also significantly improved blastocyst quality by increasing the total cell number and decreasing the percentage of TUNEL positive cells. These results indicate that E-64 supplementation during IVM is a promising tool to improve the efficiency of OPU-IVF program by improving the developmental competence of OPU-derived immature oocytes.

## 1. Introduction

In the livestock industry, assisted reproductive technologies (ARTs) have been developed to improve the reproductive efficiency and the genetic properties of animals. Among ARTs, in vitro embryo production (IVEP) emerged as the most widely promising approach in the past decade. IVEP following the recovery of female gametes from slaughtered animals has the advantage to maximize the rapid expansion of animal population, obtain a large number of zygotes at a low cost, and improve the genetic quality of the produced embryos. However, one of the main limitations is the impossibility to repeat the recovery of oocytes from the animal of high genetic merit. Therefore, the recovery of cumulus oocyte complexes (COCs) from live animals using the ovum pick-up (OPU) technique overcomes this limitation and offers an alternative approach to produce a large number of offspring with respect to the potential oocyte population contained in the ovary [1].

The first ultrasound-guided transvaginal OPU in cows was performed in 1988 by Pieterse et al., followed by many attempts to improve its efficiency [2,3,4,5,6]. The effectiveness of OPU depends on the recovery rate, which is affected by several factors, such as hormonal super-stimulation of animals, needle type, puncture frequency, time of puncture within the estrous cycle, aspiration vacuum, and the operator’s experience [7,8,9,10,11]. Despite all the progress that has been made in OPU technique, the percentage of in vitro produced blastocysts is still low (~25–35%) [12]. One of the primary reasons for the reduced rate of in vitro blastocyst production is the reduced quality and the lack of developmental competence of the recovered oocytes after fertilization [12]. Indeed, OPU-derived oocytes exhibit lower morphological quality when compared to those collected by aspiration from slaughterhouse ovaries [13]. For example, the average percentage of grade 1 oocytes per collection was ~20% for OPU-derived COCs compared to 49% for COCs collected from slaughterhouse ovaries [12,13]. Therefore, improving the culture condition necessary to promote the quality and the developmental competence of OPU-derived COCs will be a prerequisite to improve the efficiency of OPU-IVF technology.

Apoptosis is considered one of the causes of impaired developmental competence of mammalian COCs [14,15,16,17]. Poor quality OPU-derived COCs have higher rates of apoptosis when compared to good quality COCs [18]. We previously demonstrated that cathepsin B (CTSB) apoptotic pathway is a marker of inferior quality COCs and inversely correlated with the developmental competence of bovine COCs and preimplantation embryos (day 2, 4, and 7 embryos) [15]. Moreover, *Ctsb* gene expression was significantly higher in poor quality bovine embryos, compared to good quality embryos [19]. Accordingly, inhibition of CTSB activity using cell-permeable inhibitor (l-trans-Epoxysuccinyl-Leucylamido-(4-guanidino) butane, E-64) improved the developmental competence of bovine oocytes and the quality of the produced blastocysts [15,19,20]. Given the poor quality and reduced developmental competence of OPU-derived oocytes, it is possible that manipulating the apoptotic pathway during in vitro maturation (IVM) using E-64 will be a promising strategy to improve the developmental rate and the quality of the OPU-derived blastocysts.

In the present study, we show that supplementation of IVM medium with E-64 can improve the developmental competence of OPU-derived immature oocytes. In addition, E-64 supplementation improves the quality of the produced blastocysts, at least partially, through down-regulating the apoptotic rate.

## 2. Methods

The study was carried out at the Kyushu-Okinawa Agricultural Research Center, NARO, Japan. All experiments were approved by the Animal Experimental Committee of Kyushu Okinawa Agricultural Research Center, NARO.

### 2.1. Chemicals

Unless otherwise specified, chemicals were purchased from Sigma-Aldrich (St. Louis, MO, USA).

### 2.2. Post-Mortem COC Collection

Pairs of ovaries from Japanese Black cows were obtained from a local slaughterhouse. The ovaries were then washed in sterile saline containing 100 IU/mL penicillin and 100 µg/mL streptomycin (Nacalai Tesque, Kyoto, Japan). COCs were aspirated from follicles with diameters ranging from 2 to 6 mm using a 19-gauge needle attached to a 10-mL syringe.

### 2.3. Transvaginal Ultrasound-Guided OPU

Transvaginal ultrasound-guided OPU was performed weekly on 4–8 year-old, healthy, pluriparous Japanese Black cows, according to Pieterse et al. [10]. Donors were not hormonally treated before OPU. Sedation of the cow was achieved by an intramuscular injection of Xyladin (Celactal, Bayer Health care, Tokyo, Japan). Caudal epidural anesthesia was performed using procaine HCl (Kawasaki-Mitaka pharmaceuticals, Kanagawa, Japan) to prevent rectal contraction. The vulva and perineal regions were thoroughly cleaned. An electronic convex probe for veterinary use (UST-9109P- 7.5 MHz, ALOKA, Japan) was used. After retraction of the uterus, the right or left ovary was placed against the head of the transducer which had been inserted into the vagina adjacent to the cervix. All 2–6 mm visible follicles were punctured and aspirated at 90 mmHg vacuum pressure. The antral follicles appeared as black round spots on the monitor of the scanner (HS-2000V, Honda electronics, Aichi, Japan), and the puncture needle (Cow ova vacuuming needle A-type, Misawa Medical Industry, Tokyo, Japan) was depicted by a white line. The diameter of the follicles was estimated by calibration on the monitor. The transducer was positioned so that the puncture line on the monitor transected the follicle to be punctured. When a follicle was positioned steadily on the puncture line, the needle was pushed through the vaginal wall until its tip became visible within the follicle. This was directly followed by aspiration of the follicular contents. After the needle had been withdrawn, it was flushed with ovum preservation medium using 5% fetal calf serum (FCS) containing lactated Ringer’s solution (the same solution was used for ovum aspiration). All steps were done using controlled temperature tubes at 38.5 °C.

### 2.4. In Vitro Maturation

Bovine COCs were washed three times in tissue cultured medium-199 (TCM-199 (Gibco, Grand Island, NY, USA) enriched with 5% FCS, follicle stimulating hormone (FSH, 0.02 IU/mL; Denka, Kawasaki, Tokyo, Japan), estradiol-17β (1 µg/mL), and gentamicin (10 µg/mL), followed by incubation in the same medium (fifty COCs/500 µL medium volume) under mineral oil at 38.5 °C for 22 h in a humidified atmosphere of 5% CO_2_ in air.

E-64 (E3132) was dissolved in phosphate-buffered saline (PBS) and then added to TCM-199 medium to a final concentration of 1 μM. The final concentration of E-64 was selected based on our previous publications [14,15,21,22]

### 2.5. In Vitro Fertilization

Two frozen semen straws (from fertility-proven bulls) were thawed in warm water (37 °C) for 20 s. Spermatozoa were then washed by centrifugation in 90% (*v*/*v*) Percoll solution (GE healthcare bio-sciences AB, Uppsala, Sweden) at 800× *g* for 10 min. The supernatant was discarded and the pellet was then diluted with IVF-100 solution (Research institute for the functional peptides, Yamagata, Japan) prior to further centrifugation at 800× *g* for 5 min. The spermatozoa pellet was then diluted again with IVF-100 to prepare the final concentration of sperm cells at 5–10 × 10^6^/mL.

The matured COCs were washed three times with IVF-100 followed by incubation in sperm cells suspension (20 oocytes/100 µL drop) covered with mineral oil. To allow fertilization, COCs were co-incubated with spermatozoa for 6 h at 38.5 °C in a humidified atmosphere of 5% CO_2_ in air according to [14,23,24,25,26].

### 2.6. In Vitro Culture

The fertilized oocytes were denuded mechanically by pipetting in Charles Rosenkran’s 1 amino acid (CR1aa) medium [27] containing 5% FCS and supplemented with essential and non-essential amino acids. Presumptive zygotes were then transferred into micro-drops of CR1aa supplemented with 5% FCS (20–30 zygotes/50 µL medium), followed by culturing for 7 days in a humidified atmosphere of 5% O_2_, 5% CO_2_ and 90% N_2_ at 38.5 °C. Cleavage and blastocyst rates were evaluated on days 2 and 7, respectively.

### 2.7. Terminal Deoxynucleotidyl Transferase Fluorescein-dUTP Nick End Labeling (TUNEL)

To evaluate the apoptotic status of the produced blastocysts, a TUNEL assay kit (In Situ Cell Death Detection Kit, Roche applied science, USA) was employed according to Balboula et al. [14]. Blastocysts were fixed in 4% (*w*/*v*) paraformaldehyde solution (pH 7.4) for 30 min followed by a brief washing in PBS containing 3 mg/mL PVP. The blastocysts were then permeabilized in PBS with 0.5% Triton X for 20 min followed by washing (twice/10 min) in PBS with 3 mg/mL PVP. To label the fragmented DNA ends, blastocysts were incubated with fluorescein–dUTP for 60 min at 37 °C. The incubated blastocysts were washed three times in PBS with 3 mg/mL PVP for 5 min each, prior to mounting onto glass slides using DAPI (Vectashield with DAPI, Vector laboratories, Burlingame, CA, USA). Fluorescence was detected on a fluorescent microscope (TE-300, Nikon, Tokyo, Japan). To detect the fluorescence of fragmented DNA ends, excitation filter of 488 nm was used. Excitation filter of 365 nm was used to observe the DNA. Counting of total cells (marked by DAPI) or TUNEL positive cells was performed manually using Image J analysis software (NIH, Bethesda, MD, USA).

### 2.8. Statistical Analysis

Each experiment was replicated at least three times. The data were expressed as means ± SEM. The statistical significance of differences was analyzed by Student *t*-test using Prism GraphPad Software (La Jolla, CA, USA). The differences of *p* < 0.05 were considered significant.

## 3. Results and Discussion

**Figure 1:**
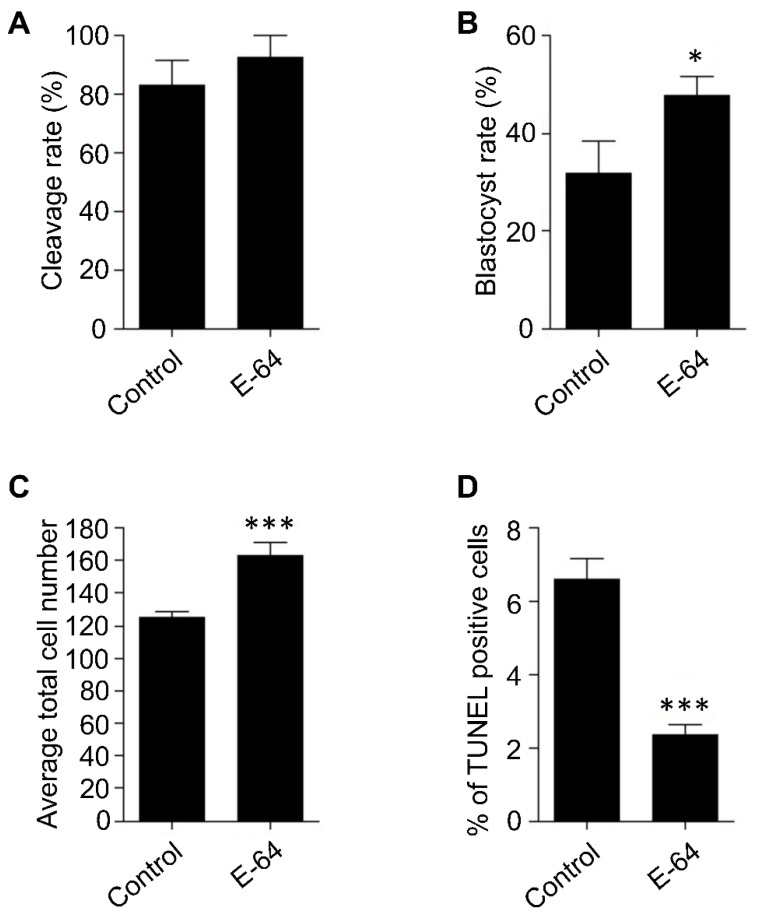
Effect of E-64 supplementation during in vitro maturation on the developmental rate and the quality of preimplantation embryos. Bovine COCs were collected separately from slaughterhouse ovaries of the same animal followed by in vitro maturation in the presence or absence of 1 μM of E-64. After in vitro fertilization, presumptive zygotes were cultured for 7 days. Cleavage (**A**) and blastocyst (**B**) rates were observed on days 2 and 7, respectively. Total number of oocytes used is 166. Total cell number of day 7 blastocysts was counted with DAPI (**C**). The apoptotic status of day 7 blastocysts was detected by TUNEL staining (**D**). Total number of embryos used is 44. The experiments were carried out at least three times. The data are expressed as mean ± SEM. Values with asterisks vary significantly, * *p* < 0.05, *** *p* < 0.001.

IVM is one of the most critical steps during IVEP, in which the oocytes acquire their developmental competence. We previously demonstrated that *Ctsb* gene expression and CTSB activity are significantly increased in poor quality mature COCs and embryos than good quality ones [15,19]. Accordingly, inhibiting CTSB activity during IVM, using E-64, significantly improved the developmental competence of bovine oocytes, most likely through regulating the apoptotic pathway [15]. OPU-derived immature COCs are notoriously prone to reduced quality [12,13], and because poor quality COCs exhibit higher rates of apoptosis, therefore, we hypothesized that CTSB regulation during IVM could be a promising tool to improve the developmental competence of OPU-derived immature oocytes. However, it is widely acknowledged that the potential of bovine embryos to develop to the blastocyst stage is highly affected by individual variations. To investigate whether CTSB inhibition during IVM has the potential to improve the developmental competence of oocytes, irrespective of individual variations, bovine ovaries were collected separately at the abattoir. Bovine COCs were aspirated from the ovaries of the same animal and then matured in vitro in the presence (E-64 group) or absence (control group) of 1 μM E-64, whose efficiency to inhibit CTSB activity was previously reported [14,15,20,28]. After IVM, COCs were in vitro fertilized and cultured for 7 days. Consistent with our previous reports [14,15], inhibition of CTSB did not affect the cleavage rate (Figure 1A), but significantly improved (*p* < 0.05) the blastocyst rate when compared to controls (Figure 1B). This improving effect of E-64 on the blastocyst rate (~47%) is comparable to several previous studies aimed to improve the efficiency of in vitro embryo production in cattle [29,30,31]. Average total cell number and the apoptotic status are considered efficient indicators for embryonic quality [32]. Inhibition of CTSB during IVM significantly increased (*p* < 0.001) the average total cell number of the resulting blastocysts (162.8 ± 8.23) when compared to controls (124.8 ± 3.62; Figure 1C). Consistent with the improving effect of E-64 on the average total cell number, inhibition of CTSB activity during IVM resulted in a significant decrease (*p* < 0.001) in the percentage of TUNEL positive cells of day 7 blastocysts (2.33 ± 0.28%) when compared to blastocysts produced from oocytes matured in E-64 free medium (Figure 1D; 6.62 ± 0.56%). The improving effect of CTSB inhibition, after excluding the genetic variation factor, suggests that CTSB inhibition could be a promising strategy to improve the developmental competence of bovine oocytes recovered from live animals using OPU technique.

**Figure 2:**
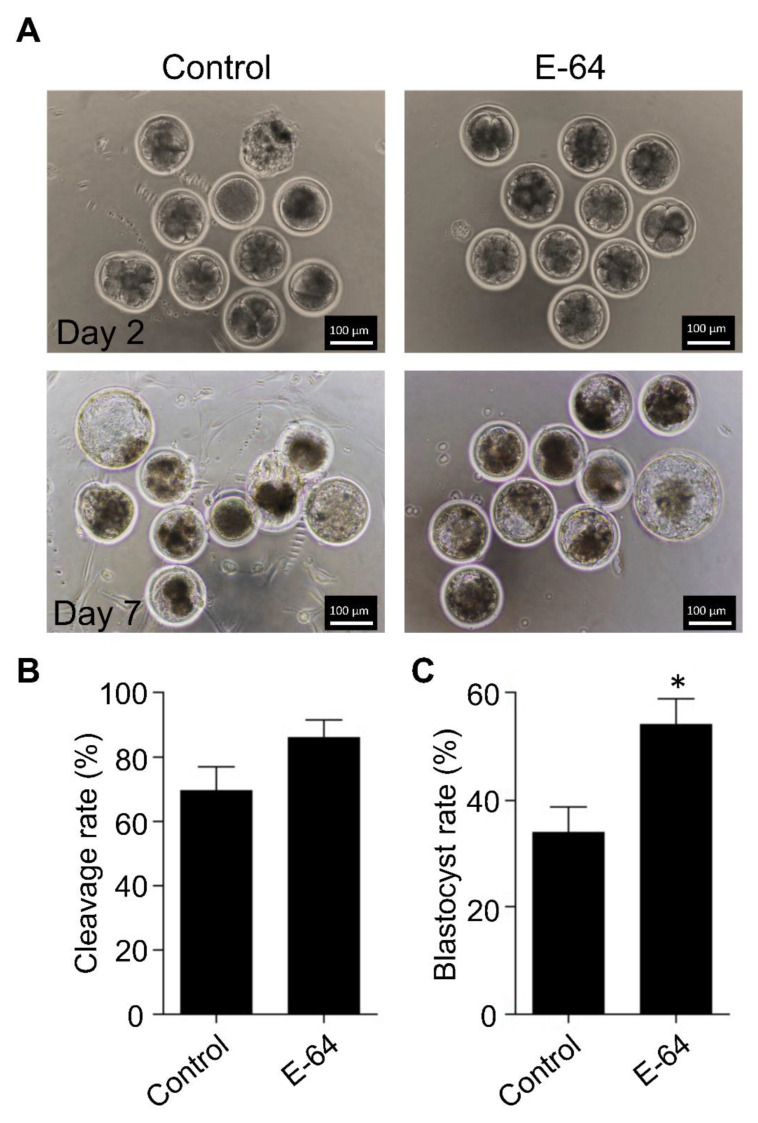
Effect of E-64 supplementation during in vitro maturation on the developmental competence of ovum pick-up-derived immature oocytes. Bovine COCs were recovered from live animals using ultrasound-guided transvaginal ovum pick-up technique. Recovered COCs were in vitro matured in the presence or absence of 1 μM of E-64. After in vitro fertilization, putative zygotes were cultured for 7 days. (**A**) Cleavage rate was evaluated on day 2 while the blastocyst rate was evaluated on day 7. Shown are representative images. (**B**) Quantifications of the cleavage rate. (**C**) Quantifications of the blastocyst rate. Total number of oocytes used is 98. The experiments were carried out five times. The data are expressed as mean ± SEM; Student’s *t*-test was used to analyze the data. Values with asterisks vary significantly, * *p* < 0.05.

**Figure 3:**
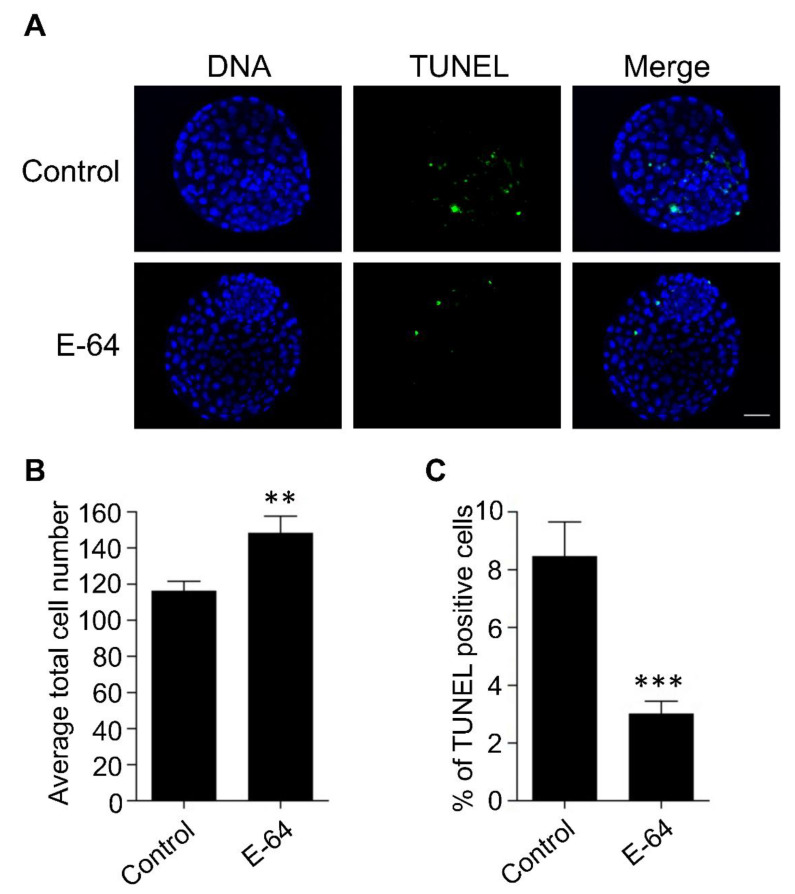
Effect of E-64 supplementation during in vitro maturation on the quality of preimplantation embryos. Bovine COCs were recovered from live animals using ultrasound-guided transvaginal ovum pick-up technique. Recovered COCs were in vitro matured in the presence or absence of 1 μM of E-64 followed by IVF and culture. (**A**) Day 7 blastocysts were examined using TUNEL staining (green) to assess the apoptotic status and DAPI (blue) to assess the total cell number. Shown are representative images. (**B**) Quantification of the average total cell number. (**C**) Quantification of the percentage of TUNEL positive cells. Total number of oocytes used is 46. The experiments were carried out three times. The data are expressed as mean ± SEM; Student’s *t*-test was used to analyze the data. Values with asterisks vary significantly, ** *p* < 0.01, *** *p* < 0.001.

To investigate the effect of CTSB inhibition on the developmental competence of OPU-derived immature oocytes, bovine COCs were recovered from live animals using transvaginal ultrasound-guided OPU technique followed by inhibition of CTSB activity during IVM. To this end, the recovered COCs were randomly distributed and in vitro matured in the presence (E-64 group) or absence (control group) of 1 μM E-64 followed by fertilization and culture for 7 days to assess cleavage and blastocyst rates. Importantly, the addition of 1 µM E-64 to the maturation medium improved the developmental rate (Figure 2A,C), as evidenced by the significant increase of the blastocyst rate (*p* < 0.05). On the other hand, no significant difference was observed regarding the cleavage rate (Figure 2A,B). In addition, inhibition of CTSB improved the quality of bovine blastocysts produced from OPU-derived immature oocytes. The total cell number of day 7 blastocysts produced from E-64-treated oocytes (147.86 ± 9.9) was significantly higher (*p* < 0.01) than that in the control group (Figure 3A,B; 116.38 ± 5.5). Moreover, supplementation of the IVM medium with E-64 significantly decreased the percentage of TUNEL positive cells in the produced blastocysts (Figure 3A,C; 2.98 ± 0.5%; *p* < 0.001) when compared to controls (8.45 ± 1.2%). To our knowledge, this is the first study that aimed to investigate the effect of CTSB inhibition on the developmental competence of OPU-derived immature oocytes in mammals. Our results of the significant increase of the developmental competence of bovine oocytes after CTSB inhibition during IVM clearly show that E-64 might be an important supplement during IVM of bovine oocytes recovered using OPU technique.

**Figure 4:**
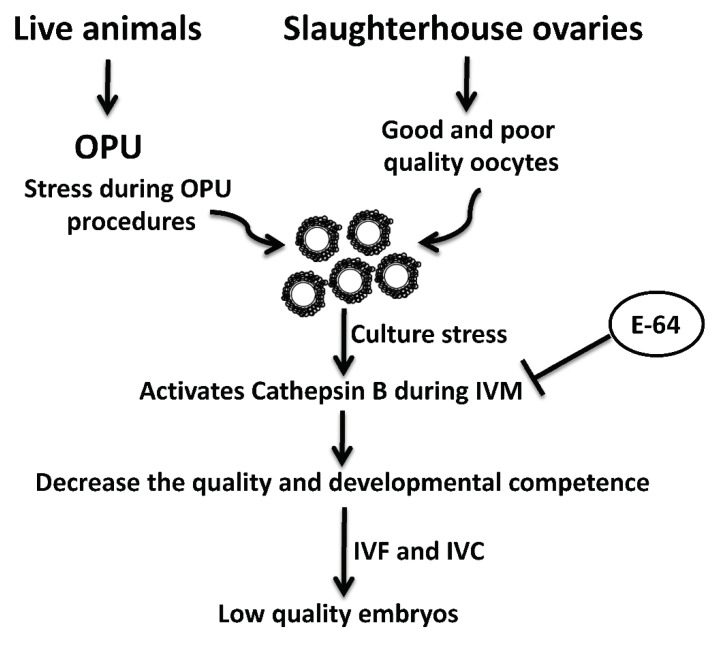
Schematic diagram of the role of cathepsin B and its inhibition by E-64 on the developmental competence and quality of bovine oocytes recovered by ultrasound-guided ovum pick-up technique.

OPU-derived COCs are notoriously prone to reduced quality [12,13]. This reduced quality might be attributed to the inappropriate vacuum pressure and needle characteristics, as well as environmental stress associated with OPU procedures and transportation [13,33,34]. Interestingly, under stress conditions, active CTSB is leaked from damaged lysosomes. Leaked CTSB protease can lead to mitochondrial membrane damage with subsequent activation of caspase 3 and initiation of the apoptotic pathway [35,36,37]. We previously demonstrated that CTSB activity is negatively correlated with the quality of bovine oocytes and preimplantation embryos. CTSB activity is significantly increased in poor morphological quality oocytes and embryos when compared to those with good quality [14,19]. Given that OPU-derived oocytes have a relatively poor quality with high rates of apoptosis [13,18], it is plausible that E-64 enhanced the developmental rate and the quality of bovine COCs by regulating the apoptotic pathway (Figure 4). A limitation of this study is that we used once-weekly OPU protocol that can allow for the development of dominant follicles which might affect the quality and stimulate apoptosis in oocytes within subordinate follicles. It will be interesting to investigate whether CTSB inhibition improves the quality of oocytes recovered by hormonal stimulation or twice-weekly OPU protocol.

## 4. Conclusions

Our findings indicate that CTSB inhibition is a promising strategy to improve the efficiency of OPU-IVF program by improving the quality and developmental competence of OPU-derived immature oocytes.

## Data Availability

All data generated or analyzed during this study are included in this published article.

## Abbreviations

ARTs	Assisted reproductive technologies
IVEP	In vitro embryo production
COCs	Cumulus oocyte complexes
OPU	Ovum pick-up
CTSB	Cathepsin B
IVM	In vitro maturation
E-64	(l-trans-Epoxysuccinyl-Leucylamido-(4-guanidino) butane)
FCS	Fetal calf serum
FSH	Follicle stimulating hormone
TCM-199	Tissue cultured medium-199
PBS	Phosphate-buffered saline
CR1aa	Charles Rosenkran’s 1 amino acid
TUNEL	Terminal deoxynucleotidyl transferase fluorescein-dUTP nick end labelling
